# Irradiated chitosan nanoparticles and biological agent: a novel approach for management of sesame wilt disease

**DOI:** 10.1007/s00425-025-04913-9

**Published:** 2026-02-05

**Authors:** Ahmed S. Fares, Naeema G. Hassan, Hala A. Mahdy, Heba E. Aboelmagd

**Affiliations:** 1 Plant Research Department, Nuclear Research Centre, Egyptian Atomic Energy Authority (EAEA), Cairo, Egypt; 2 Plant Pathology Department, Faculty of Agriculture, Benha University, Benha, Egypt; 3 Plant Pathology Department, Institute of Agriculture Research Center, Giza, Egypt

**Keywords:** *Fusarium oxysporum* f. sp. *sesami*, Chitosan nanoparticles, *Trichoderma reesei*, Biological control, Gamma irradiation

## Abstract

These results highlight irradiated Ch-NPs as a promising, eco-friendly, and sustainable component of integrated disease management strategies, offering a viable alternative to conventional chemical fungicides for controlling Fusarium oxysporum f. sp. sesami in sesame cultivation.

Fusarium wilt, caused by *Fusarium oxysporum* f. sp. *sesami*, poses a major limitation to sesame productivity. To develop a more efficient control approach, chitosan nanoparticles (Ch-NPs) were synthesized and exposed to 24 kGy of gamma irradiation, a process that improves their structural uniformity and enhances their functional properties. The antifungal activity was tested under laboratory, greenhouse, and field conditions, either alone or in combination with *Trichoderma reesei*, *Bacillus subtilis*, and the commercial fungicide Maxim-XL. The nanoparticles were characterized using UV, FTIR, and TEM analysis. UV analysis confirmed the nanoparticle spectrum with a maximum absorbance at 224 nm. Using transmission electron microscopy, it was found that by gamma irradiation, Ch-NP size was reduced from 89.08–113.63 nm to 48.11–56.22 nm, which, in turn, made it more uniform and bioactive. Irradiated Ch-NPs (250 µL L⁻^1^) demonstrated the ability of almost complete inhibition of *F. oxysporum* growth in vitro, along with controlling the disease incidence and severity in greenhouse and field tests, which is equal to that of Maxim-XL. Among the biological agents tried, *T. reesei* was the best in giving an antagonism of 76.3% inhibition. Treatment with irradiated Ch-NPs and *T. reesei* enhanced sesame growth and productivity, reflected in greater plant height, more capsules, and higher seed yield, and also elevated the activities of defense-related enzymes—peroxidase, polyphenol oxidase, chitinase, and phenylalanine ammonia-lyase. The study therefore sought to assess the effectiveness of gamma-irradiated chitosan nanoparticles, used in combination with biological control agents, as eco-friendly alternatives to chemical fungicides for managing the disease.

## Introduction

Sesame (*Sesamum indicum* L.) contains several vitamins, minerals, antioxidants, and beneficial fatty acids; the oil is considered safe for consumption. It is widely cultivated in the tropical and subtropical regions throughout the world (Debnath et al. 2025). Despite its significance, sesame production is severely constrained by several diseases, with Fusarium wilt—caused by *Fusarium oxysporum* f. sp. *sesami*—representing one of the most destructive threats. This soil-borne pathogen can infect plants from germination to full maturity and causes significant yield loss (Ngamba et al. 2020; Mahdy et al. 2022). Several management measures have been investigated to control Fusarium wilt, including crop rotation, resistant varieties, and chemical fumigation. While the use of resistant varieties presents a sustainable control option, the unabated emergence of novel races of the pathogen that break down host resistance continues to be a major setback (Zhang et al. 2020; Lal et al. 2024). More than that, the use of conventional chemical fungicides remains risky to the environment and places a metabolic expense on the finances of the peasant farmer. Consequently, for the past years, more efforts have focused on finding effective, inexpensive, and environmentally friendly alternatives. The breakthroughs in the application of nanobiotechnology and biocontrol represent the potential plant disease management tools for the future. The combination of the use of nanomaterials(Fares et al. 2024a, 2024b), such as chitosan nanoparticles, and the beneficial microbial agents has the potential of stimulating plant defensive processes and suppressing phytopathogens in the most environmentally clean way possible (Ouda 2014; Zaman et al. 2025; Manimaran et al. 2025). These activation processes increase the physical and biochemical protection of the plant, including the activation of major enzymes, e.g., phenylalanine ammonia-lyase (PAL), peroxidase, and chitinase, culminating in resistance to the pathogen (Hura et al. 2025). Chitosan effects on plant root systems and its potential application in managing fungal diseases in agriculture were studied (Suwanchaikasem et al. 2024). Chitosan, a biopolymer derived from chitin, exhibits notable antifungal properties while remaining biodegradable, biocompatible, and non-toxic. Owing to these attributes, it has been widely applied in managing fungal phytopathogens. The antifungal efficacy of chitosan can be enhanced considerably when it is engineered into nanoparticle form (Poznanski et al. 2023). Chitosan nanoparticles (Ch-NPs), recognized for their positive charge and polymeric composition, have been explored in recent scientific work (El-Naggar et al. 2022). Chitosan nanoparticles have promising biophysicochemical properties such as high surface area, positive surface charge, and functional group availability, creating potential application in agriculture. Chitosan and promoting bacteria (PGPB) are promising biotechnological tools in modern agriculture to enhance plant growth-promoting bacteria. Their combined application offers an opportunity to develop production systems that minimize harmful ecological and health effects while enhancing plant growth, stress, and overall productivity (Azevedo et al. 2024). Ch-NPs, due to their biocompatibility, lesser toxicity, and ability to penetrate plant cells, contribute to their potential use as a deliverer of biocontrol agents for disease control (Wang et al. 2024; El-Saadony et al. 2025). Characterization of nanoparticles using multiple analytical techniques such as UV–visible spectroscopy, Fourier transform infrared spectroscopy (FTIR), X-ray diffraction (XRD), and scanning electron microscopy (SEM) coupled with energy-dispersive X-ray (EDX) analysis is essential to confirm their structural, compositional, and functional properties, which directly relate to their biological activity and performance in plant disease management (Carrillo-Lopez et al. 2024). Even though Ch-NPs have promising potential, they have, however, failed to draw major attention in plant pathology, especially in the field of fungal disease control. In parallel, microbial biocontrol agents such as *Trichoderma* spp., *Bacillus* spp., and *Pseudomonas fluorescens* manifested high antagonism activity against soil-borne phytopathogens and the potential to improve plant growth. *Trichoderma* species have the ability to suppress pathogenic fungi as well as induce host defense systems, whereas the antifungal activity of *Bacillus subtilis* results from the production of antibiotics and lytic enzymes. In the same way, *Pseudomonas fluorescens* has extensively been reported to suppress the infections related to the roots, including the wilt diseases (Abdelrhim et al. 2023; Maral-Güle & Eltem 2025; Madlhophe et al. 2025). This study evaluates the potential of gamma-irradiated chitosan nanoparticles, alone and in combination with *Trichoderma reesei*, *Bacillus subtilis*, and a commercial fungicide, in the control of Fusarium wilt of sesame under laboratory, greenhouse, and field conditions. The study equally touches on associated physiological activity and defense enzyme activity in a quest to identify disease-suppressive mechanism. In general, this work represents a novel dual-action approach, integrating gamma-irradiated chitosan nanoparticles and a biological control agent, to effectively control sesame wilt in both controlled and field environments.

## Materials and methods

### Isolation of the pathogen

Infected sesame samples were collected from Qalyubia Governorate, Egypt. Under running tap water, the roots and stems were cleaned and cut into segments of 0.5–1 cm. Three minutes of 1% sodium hypochlorite surface sterilization was followed by four sterile distilled water (SDW) rinses. Plated onto Fusarium selective medium augmented with streptomycin sulfate (0.4 g/L), samples were dried on sterile filter paper. Incubation was carried out at 28 ± 2 °C for 72 h, followed by purification of fungal isolates using single spore and hyphal tip techniques, and cultured and maintained at 5 °C on potato dextrose agar slants, with subculturing every 2 weeks. The source of the bio-agent *Trichoderma reesei* (AUMC13026) was collected from the Faculty of Agriculture, Mansoura University, and *Bacillus subtilis* was provided by the Plant Pathology Research Institute, Agricultural Research Center, Giza, Egypt.

### Pathogenicity test

Fusarium isolate pathogenicity was tested on sesame plants through controlled greenhouse conditions. Healthy seedlings in the 3–4 leaf stage were carefully removed from soil and washed with sterile distilled water. The root-dip method was followed by inoculation of the seedlings into conidial suspension of the Fusarium isolate (1 × 10^6^ conidia mL^−1^) for 30 min. Control plants were treated in the same way with sterile distilled water. Seedlings were inoculated and maintained in sterilized soil under 25 ± 2 °C temperature and 70% relative humidity in natural light conditions. Disease development was monitored for 21 days post-inoculation. Inoculated plants exhibited normal symptoms of Fusarium wilt, including yellowing of the leaves, browning of the vasculature, and stunted growth, whereas control plants were normal. To confirm Koch’s postulates, the pathogen was re-isolated from infected tissues and identified morphologically and molecularly, which confirmed its role as the inoculated Fusarium isolate.

### Plant material

Seeds of sesame (*Sesamum indicum* L., cultivar ‘Shandawil 3’) were obtained from the Oil Crops Research Department, Agricultural Research Center, Egypt. Prior to use, the seeds were surface sterilized with 1% sodium hypochlorite for 2 min and rinsed three times with sterile distilled water (SDW).

### Fungicide treatment

Maxim-XL, a commercial fungicide formulation containing fludioxonil (1.5%) and metalaxyl (2%), was obtained from Syngenta Agro, Egypt. It was applied to sesame seeds at the recommended label rate as a chemical control treatment.

### Preparation of chitosan nanoparticles (Ch-NPs)

Low molecular weight chitosan was provided by Sigma-Aldrich Germany, while the sodium tripolyphosphate (STPP) was provided. Budi et al. (2020) developed chitosan nanoparticles through ionic gelation by applying modified techniques to the original method. Briefly, chitosan was dissolved in 1% acetic acid at a concentration of 0.2 g/100 mL and stirred at 1300 rpm for 2 h at room temperature. The process of nanoparticle formation started after 0.066 g/100 mL STPP solution was added dropwise to the continuously stirred solution. The nanosuspension solution needs to be stored at 4 °C until it becomes necessary for usage.

### Characterization of nanoparticles

Transmission electron microscopy (TEM) was used to characterize the morphology and size distribution of the irradiated Ch-NPs. Imaging was conducted using a JEOL JEM-2100, FTIR and U.V. spectrophotometer which were instruments at the National Research Center, Egypt.

### Gamma irradiation of Ch-NPs

The prepared chitosan nanoparticles were exposed to 24 kGy of gamma irradiation using a cobalt-60 source housed at the Cyclotron Facility, Nuclear Research Centre, Egyptian Atomic Energy Authority, Egypt.

### Identification and sequencing of Fusarium oxysporum f. sp. sesami.

This strain has been deposited at Assiut University Mycological Center (AUMC), Egypt, under strain number AUMC 17117.

### Preparation of bio-agent inocula

*Trichoderma reesei* was first cultured on potato dextrose agar and incubated at 25 °C for 7 days to ensure that the fungus would grow vigorously. Spores were harvested by washing the colony surface gently with sterile distilled water, and filtration was used to eliminate any remaining mycelia. The suspension was diluted to reach a concentration of 1 × 10⁶ spores mL^−1^ and quantified using a hemocytometer. Meanwhile, *Bacillus subtilis* cultures were grown in nutrient broth at 28 °C with shaking at 150 rpm for 48 h until late log phase to ensure that bacteria are metabolically active. The cultures were adjusted to 1 × 10⁹ CFU/mL and used.

### In vitro antagonism assay

*Fusarium oxysporum* was evaluated using the dual culture method for the antagonistic activity of *Trichoderma reesei* and *Bacillus subtilis* against (Zlata et al. 2008). A 5 mm agar disc of actively growing *F. oxysporum* was placed 1 cm from the edge of a PDA plate. On the opposite side, a 5 mm disc of *T. reesei* or a loop of *B. subtilis* culture was inoculated. Plates were incubated at 27 °C for 7 days. Control plates were inoculated with the pathogen only. The inhibition rate (%) was calculated using the following formula:

$${\text{Inhibition rate }}\left( \% \right) \, = \, \left[ {\left( {{\text{R }} - {\text{ r}}} \right)/{\mathrm{R}}} \right] \, \times { 1}00$$

where R is the radial growth of *F. oxysporum* in the control and **r** is the radial growth in the presence of the antagonist (Lamsal et al. 2011).

### Greenhouse experiments

The plastic pots (30 cm across) were first soaked in a 5% formalin solution for 15 min, and then air-dried for 24 h. Each pot was filled with a sterilized soil mix of clay and sand at a 2:1 ratio, which had been autoclaved at 121 °C for 30 min on consecutive 2 days. *Fusarium oxysporum* inoculum was cultured at 27 ± 2 °C for 14 days until the culture was fully infected with fungus. Then, the fungus-infected sand and barley samples were thoroughly blended with the sterilized soil at 2% (w/w) and left sitting for 48 h to allow the mixture to mature before planting. Sesame seeds were surface-sterilized using a 1% sodium hypochlorite solution for 2 min and afterwards thoroughly rinsed with sterile distilled water, and then planted on the fungus-infected soil using the following treatment protocols: (1) water irrigation with chitosan nanoparticles (Ch-NPs) treatment of the soil at the fixed concentrations, (2) treatment using the biological control agent, both applied on the seeds and introduced into the soil, (3) treatment combining the use of the biological agent and the Ch-NPs, and (4) the untreated control. The experimental design used the completely randomized design model (CRD) layout that involved three repetitions per treatment, each of which contained three plants. The experimental setup used greenhouse conditions with a laboratory supply of controlled environmental parameters, that is, 28 ± 2 °C, 70–75% relative humidity, and a 12-h light period. In addition, the plants were watered when needed so that the soil moisture was optimal. Both the disease incidence and disease intensity on the plants were measured using the wilt rating system a month following the initial planting.

### Treatment details, experimental replications and yield parameter

Each treatment was arranged in a randomized complete block design (RCBD) with three replications, and each plot measured 3.2 × 2.4 m, containing three rows of 60 cm spacing. The test pathogen inoculum (*F. oxysporum*) was incorporated into the soil at 10⁶ CFU g⁻^1^ 1 week before sowing. The treatments received application during sowing as well as 25 days following sowing through foliar spray of nanoparticle and bio-agent formulations. The plant height together with the number of capsules per plant and the seed production per plant served as the yield measurement.

(T1): Infected control (*Fusarium oxysporum* f. sp*. sesami*).

(T2): Chitosan nanoparticles (Ch-NPs, 250 µL L⁻^1^).

(T3): Chitosan nanoparticles (Ch-NPs, 500 µL L⁻^1^).

(T4): Chitosan nanoparticles (Ch-NPs, 1000 µL L⁻^1^).

(T5): Ch-NPs + gamma-irradiated + 24 kGy (250 µL L⁻^1^).

(T6): Ch-NPs + gamma-irradiated + 24 kGy (500 µL L⁻^1^).

(T7): Ch-NPs + gamma-irradiated + 24 kGy (1000 µL L⁻^1^).

(T8): Chitosan 250 mg/L.

(T9): Chitosan 500 mg/L.

(T10): Chitosan 1000 mg/L.

(T11): *Trichoderma reesei* (1 × 10⁶ spores mL⁻^1^).

(T12): *Bacillus subtilis* (1 × 10⁹ CFU mL⁻^1^).

(T13): Chemical fungicide Maxim-XL (3.5%, recommended dose).

(T14): Healthy control (uninoculated).

### Disease assessment and plant growth evaluation

Fusarium wilt symptoms were evaluated 30 days after pathogen inoculation. Disease incidence (DI%) and disease severity (DS%) were recorded using the 0–5 disease scale where 0 = healthy and 5 = completely wilted or dead plant. Disease severity was calculated using the following formula:

$${\mathrm{DS}}\% \, = \, \left[ {\Sigma \left( {{\text{n }} \times {\text{ v}}} \right)/\left( {{\text{X }} \times {\text{ N}}} \right)} \right] \, \times { 1}00$$

where n is the number of plants showing a given severity rating, v is the severity score (0–5), X is the maximum possible rating (5), and N is the total number of plants assessed (Moharam and Negim, 2012).

Plant growth parameters, including plant height, number of capsules per plant, seed yield per plant, were also recorded at the same time point.

### Enzyme activity assays

Enzymatic activity was assessed in fresh leaf tissues 30 days post-inoculation. Peroxidase (POD) activity was measured by the guaiacol oxidation protocol of Hammerschmidt et al. (1982). Each 3.0 mL reaction contained 2.7 mL of 10 mM sodium phosphate buffer (pH 6.0), 100 µL of 0.25% guaiacol, 100 µL of enzyme extract, and 100 µL of 10 mM H₂O₂. Absorbance changes at 470 nm, reflecting tetraguaiacol production, were measured to calculate POD activity.

### Polyphenol oxidase (PPO) activity

We determined polyphenol oxidase (PPO) activity followed a slightly modified method described by Zavarise et al. (2025). Briefly, in 5 mL of sodium phosphate buffer (0.1 M, pH 6.8) containing 1% polyvinylpyrrolidone, we homogenized 1 g of fresh leaf tissue; then, we centrifuged this homogenate at 12,000 rpm for 20 min at 4 °C, and the supernatant was used as the enzyme extract. PPO activity was quantified by recording the change in absorbance at 420 nm for 3 min.

### Chitinase activity

Chitinase activity was assayed according to the colorimetric method of Reid and Ogrydziak (1981) with slight modifications. The reaction mixture (1.0 mL) containing 50 mM sodium acetate buffer (pH 5.0) and 0.5% (w/v) colloidal chitin was performed in 500 μL of enzyme extract, and other components above identical amounts of enzyme were used with appropriate reactions from the reference incubations. The reaction was kept at 37 °C for 60 min and interrupted by adding 1 mL of dinitrosalicylic acid (DNS) reagent. The tubes were then boiled for 5 min and cooled to room temperature. The absorption of the colored solution obtained was measured spectrophotometrically at 540 nm.

### Phenylalanine ammonia-lyase (PAL)

Phenylalanine ammonia-lyase (PAL) activity was determined following the procedure described by Dickerson et al. (1984), with minor modifications. Fresh plant tissue (1 g) was homogenized in 5 mL of 50 mM borate buffer (pH 8.8) and centrifuged at 12,000 × g for 15 min at 4 °C. The supernatant was used as the enzyme extract.

### Statistical analysis

All data were subjected to analysis of variance (ANOVA) using the procedures. Treatment means were compared using the least significant difference (LSD) test at the 5% probability level. All assumptions of ANOVA (normality and homogeneity of variance) were checked before analysis. Statistical computations were performed using CoStat software (version 6.45, Software, USA).

## Results

### Identification and sequencing of Fusarium oxysporum f. sp. sesami.

**Fig. 1:**
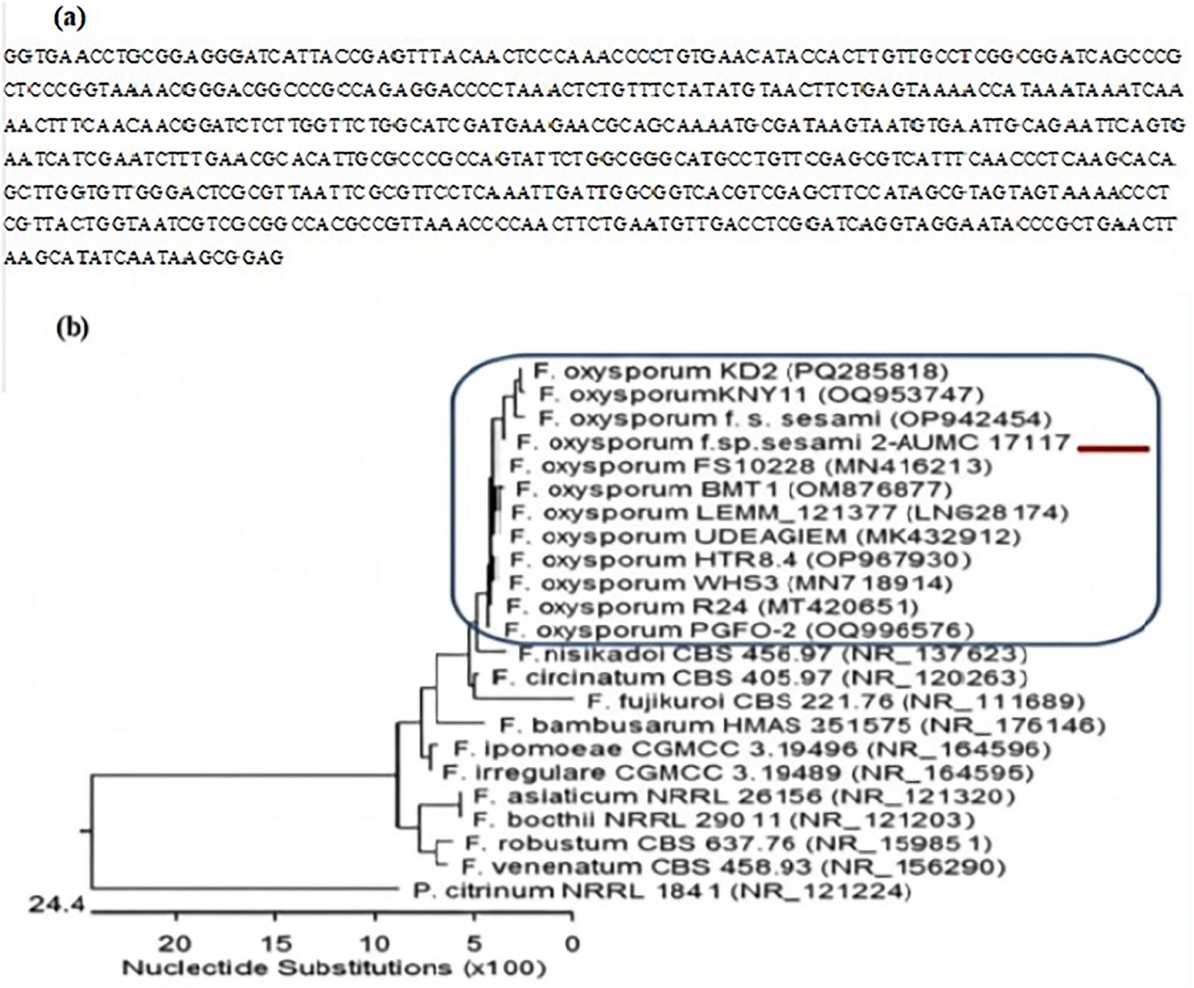
**a, b** Molecular identification and sequencing of the pathogenic *Fusarium oxysporum* f. sp. *sesami* isolate. **a** Nucleotide sequence of the internal transcribed spacer (ITS) region amplified from the pathogenic isolate recovered from diseased sesame plants. The obtained sequence was used for molecular fingerprinting and showed 100% identity and complete query coverage with reference *F. oxysporum* f. sp. *sesami* sequences available in GenBank, confirming the pathogen’s taxonomic identity. **b** Phylogenetic tree based on ITS sequences of rDNA of the fungal strain isolated in the present study (Fusarium oxysporum f. sp. sesami isolate 1 strain AUMC 17117, arrowed) aligned with closely related strains accessed from the GenBank.

A pathogenic isolate of the *Fusarium oxysporum* f. sp. *sesami* fungus was isolated from naturally infected sesame crops and identified by a combination of morphological identification and molecular identification (Fig. 1a and b). This isolate had several morphological characteristics of the *F. oxysporum* fungus and also had these characteristics further ratified by molecular analysis by the sequence of the ITS regions. This ITS sequence had 100% identity and coverage with several isolates of the *F. oxysporum* f. sp. *sesami* pathogen deposited in GenBank (Fig. 1a). Phylogenetic analysis of the ITS rDNA sequence situated the recovered isolate *F. oxysporum* f. sp. *sesami* isolate, strain AUMC 17117, aligned with *F. oxysporum* isolates and different from other species with *Penicillium citrinum* as an outgroup (Fig. 1b). The sequence of the identified strain has been deposited in the GenBank database with the accession number PX496002 and can be accessed through this link: https://www.ncbi.nlm.nih.gov/nuccore/PX496002.

### Effect of irradiated chitosan nanoparticles on Fusarium wilt severity in sesame under field conditions

**Fig. 2:**
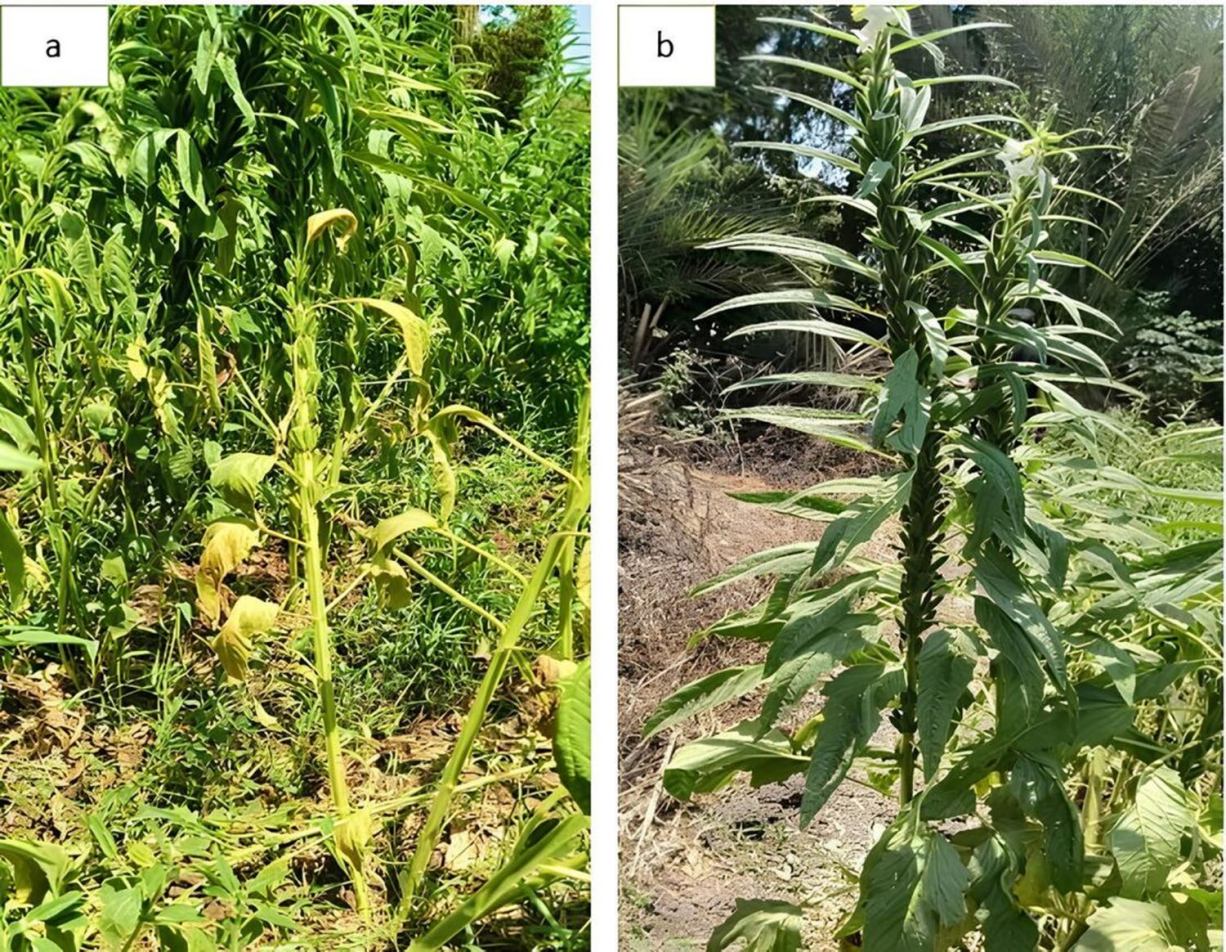
**a, b** Field symptoms of *Fusarium oxysporum* f. sp. *sesami* on sesame plants and the protective effect of irradiated chitosan nanoparticles. **a** Untreated, infected sesame plant showing typical wilt symptoms, including yellowing, necrosis, and severe stunting. **b** Sesame plant treated with irradiated chitosan nanoparticles at 24 kGy exhibiting healthy growth and normal capsule development under natural infection conditions

Field observations revealed a marked difference in disease expression between infected sesame plants and those treated with chitosan nanoparticles. The symptoms exhibited by the infected plants included progressive yellowing of lower leaves, necrosis of leaves, drooping of leaves, severe vascular discoloration, and stunted growth and collapse (Fig. 2a). Plants treated with irradiated chitosan nanoparticles retained their normal morphology and robust, healthy green foliage and upright stems, with continuing formation of capsules (Fig. 2b). The visible improvement in plant vigor confirms the protective effect of chitosan nanoparticles, which suppressed pathogen development and minimized symptom expression under field conditions.

### Morphological and cultural characteristics of Fusarium oxysporum f. sp. sesami

**Fig. 3:**
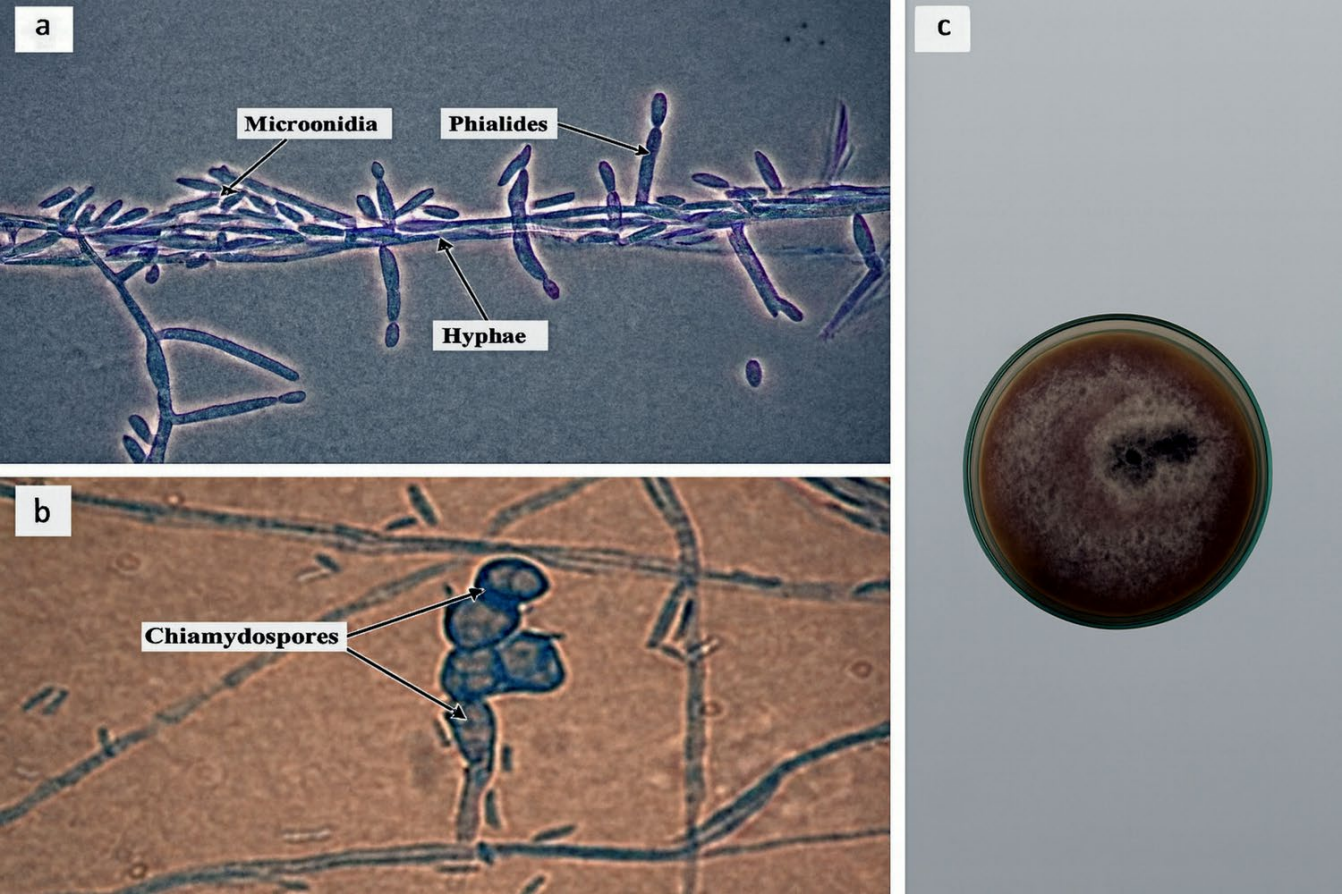
**a, b**, and **c** Morphological and cultural features of *Fusarium oxysporum* f. sp*. sesami.* **a** Septate hyphae bearing phialides and abundant microconidia. **b** Thick-walled chlamydospores observed as survival structures. **c** Colony morphology on PDA showing dense, cottony mycelial growth typical of the *Fusarium oxysporum* species complex.

Microscopic examination of the isolated pathogen confirmed morphological features characteristic of *Fusarium oxysporum* f. sp. *sesami*. Under light microscopy, the hyphae appeared hyaline and septate, bearing abundant short phialides that produced chains of ellipsoidal to oval microconidia (Fig. 3a). In addition, the fungus produced thick-walled chlamydospores, either singly or in pairs, which serve as long-term survival structures in soil (Fig. 3b). On PDA medium, the isolate formed dense, cottony mycelial growth with a pale to pinkish coloration and a slightly raised colony center typical of the *F. oxysporum* species complex (Fig. 3c). These combined morphological and cultural characteristics confirmed the identity of the pathogen as *F. oxysporum* f. sp. *sesami*.

### Characterization of chitosan nanoparticles (Ch-NPs)

#### UV–Vis characterization of chitosan nanoparticles

**Fig. 4:**
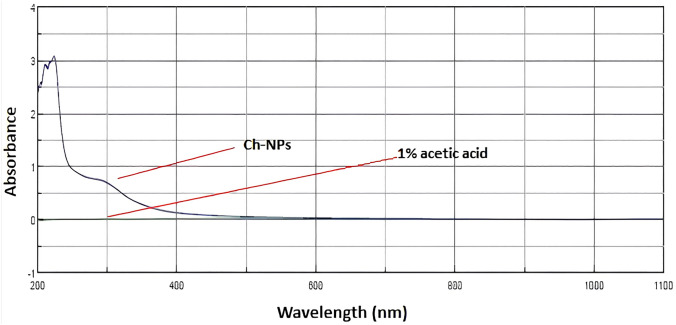
UV–Vis absorption spectrum of chitosan nanoparticles (Ch-NPs) compared with the 1% acetic acid blank. The Ch-NPs exhibited a broad absorption band within 200–350 nm, corresponding to the characteristic electronic transitions of chitosan nanostructures. The 1% acetic acid solution, used as a baseline, showed no notable absorbance across the measured wavelengths. The distinct peak of Ch-NPs confirms successful nanoparticle formation and optical stability in the acidic medium

The UV–Vis spectra of chitosan nanoparticles (Ch-NPs) as shown in Fig. 4 exhibited a prominent absorption peak within the 200–350 nm range, indicative of electronic transitions in the chitosan backbone and validating the successful synthesis of nanoparticles. Conversely, the standard solution of 1% acetic acid exhibited a nearly flat baseline, signifying minimal absorbance in this region. The absorption peak detected in the chitosan nanoparticles results from electronic transitions in the amino and hydroxyl groups of the chitosan framework, indicating maintained structural integrity post-curing and demonstrating successful synthesis and stability of the nanoparticles.

#### Fourier transform infrared spectroscopy (FTIR) spectrum of synthesized chitosan nanoparticles

**Fig. 5:**
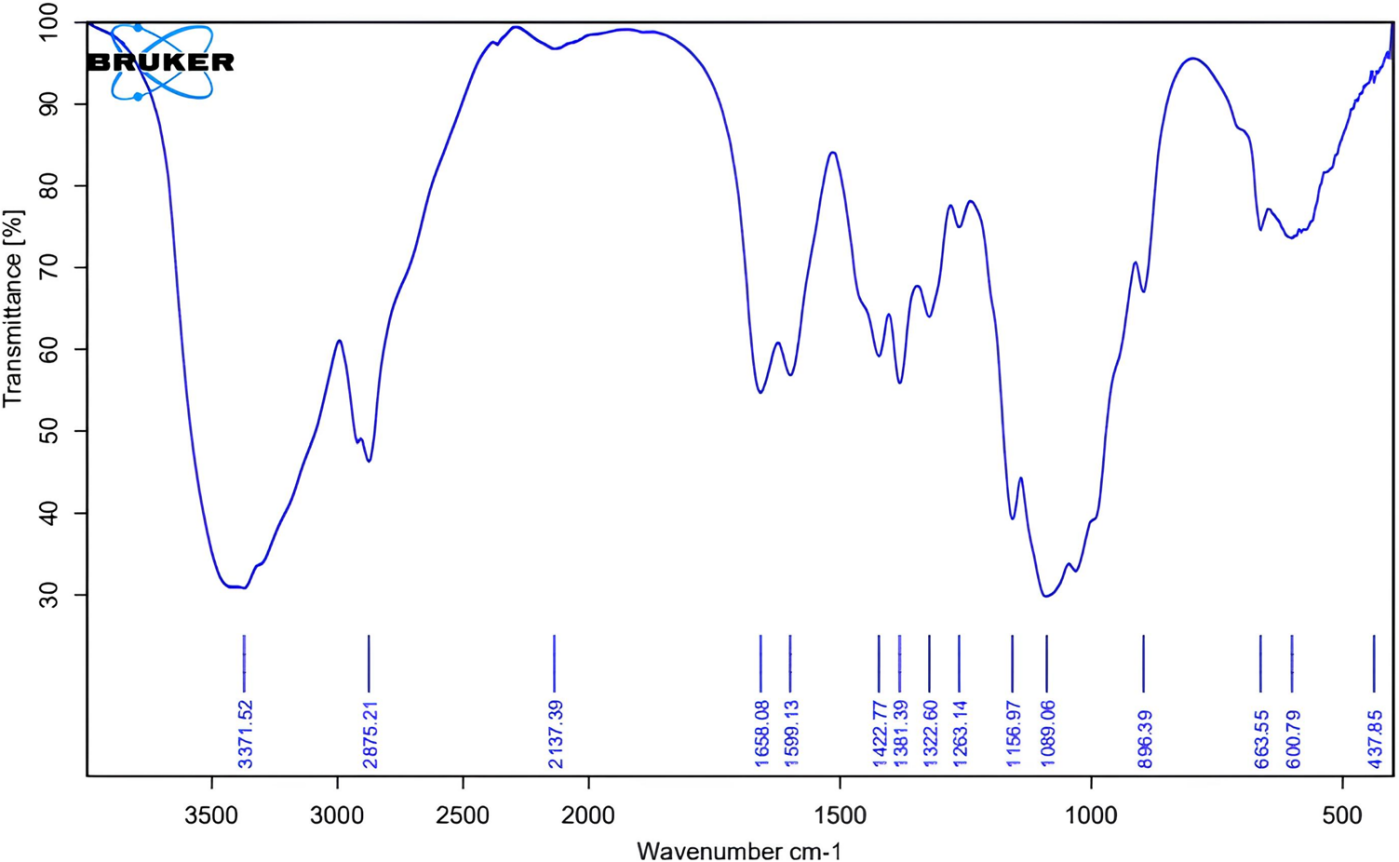
The FTIR spectrum of the synthesized chitosan nanoparticles shows the characteristic functional groups of chitosan. The broad band at 3400–3200 cm⁻^1^ corresponds to O–H and N–H stretching vibrations, while peaks at 2920–2850 cm⁻^1^ reflect aliphatic C–H stretching. Absorptions near 1650–1580 cm⁻^1^ indicate amide-related vibrations associated with residual acetamide groups. Signals at 1450–1380 cm⁻^1^ represent C–H bending, and strong peaks in the 1150–1020 cm⁻^1^ region correspond to C–O–C and C–O stretching of the polysaccharide backbone. The fingerprint region (900–500 cm⁻^1^) displays characteristic glucosamine ring vibrations, confirming the structural integrity of chitosan after nanoparticle synthesis

The FTIR spectrum displays the characteristic functional groups of chitosan nanoparticles. The O–H and N–H stretching vibrations create a broad strong band in the range of 3400–3200 cm⁻^1^ which confirms the presence of hydroxyl and amino groups. The peaks between 2920 and 2850 cm⁻^1^ show the C–H stretching vibrations that belong to aliphatic hydrocarbons. Two distinct peaks between 1650 and 1580 cm⁻^1^ in the FTIR spectrum indicate the presence of remaining acetamide groups in chitosan through amide I and amide II vibrations. The spectrum shows C–H bending through additional peaks, which appear between 1450 and 1380 cm⁻^1^. The bands at 1150–1020 cm⁻^1^ show the C–O–C bridge stretching together with C–O stretching, which forms the polysaccharide backbone. The 900–500 cm⁻^1^ region of the spectrum shows signals that represent the glucosamine ring’s skeletal vibrations. The nanoparticle formation process preserves the chitosan structure according to these signals (Fig. 5).

#### Transmission electron microscopy (TEM)

**Fig. 6:**
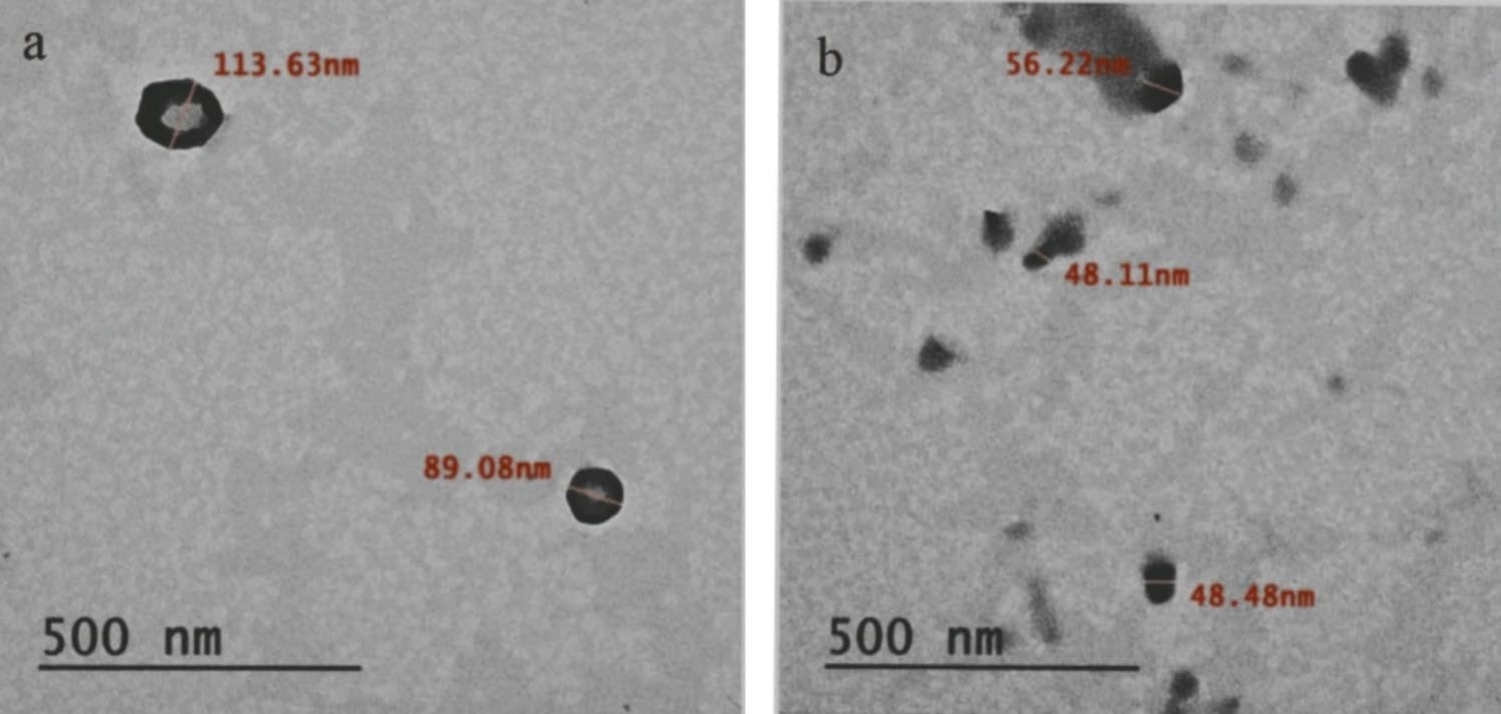
a and b Transmission electron microscopy (TEM) indicates that there are considerable differences in the distribution of particle sizes between chitosan nanoparticles that were not subjected to gamma irradiation and those that were. **a** The average size of Ch-NPs was between 89.08 and 113.63 nm. **b** The size range of the particles irradiated with a dose of 24 kGy was notably reduced to 48.11–56.22 nm

Gamma radiation at 24 KGy has played a beneficial physical agent in decreasing and enhancing the size of Ch-NPs as compared to Ch-NPs without radiation (Fig. 6 a, b). It was observed under transmission electron microscopy (TEM) that there are considerable differences in the distribution of particle sizes between chitosan nanoparticles that were not subjected to gamma irradiation and those that were. The average size of Ch-NPs was between 89.08 and 113.63 nm (Fig. 6a), while the size range of the particles irradiated with a dose of 24 kGy was notably reduced to 48.11–56.22 nm (Fig. 6b). This reduction in particle size indicates that gamma irradiation acted as an effective physical modifier, enhancing nanoparticle dispersion and uniformity.

#### Effect of chitosan, chitosan nanoparticles (Ch-NPs), and irradiated Ch-NPs (Ch-NPs + 24 kGy) on the mycelial growth of Fusarium oxysporum f. sp. sesami (FOS)

**Fig. 7:**
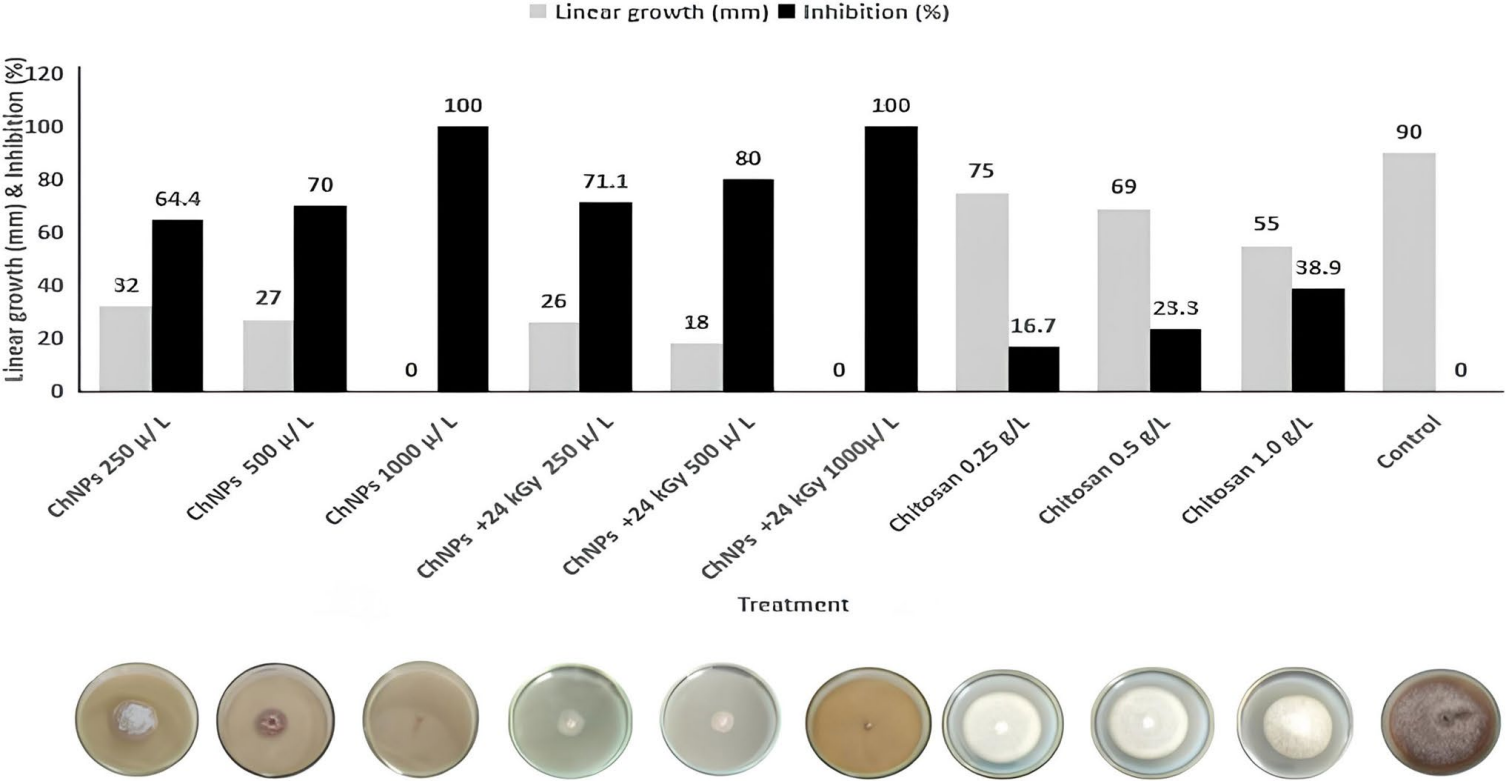
Effect of chitosan, chitosan nanoparticles (Ch-NPs), and gamma-irradiated chitosan nanoparticles (Ch-NPs + 24 kGy) on the mycelial growth of *Fusarium oxysporum* f. sp. *sesami* (FOS). Linear growth (mm) and inhibition percentage of FOS were assessed after treatment with Ch-NPs (250, 500, and 1000 µL L⁻^1^), gamma-irradiated Ch-NPs at 24 kGy (250, 500, and 1000 µL L⁻^1^), and native chitosan (250, 500, and 1000 mg L⁻.^1^)

The antifungal activity of chitosan-based treatments was tested through multiple concentration levels (250, 500, and 1000 µL/L for Ch-NPs; 250, 500, and 1000 µL/L for irradiated Ch-NPs; and 250, 500, and 1000 mg/L for non-nano chitosan) against *F. oxysporum* f. sp. *sesami*. The fungal growth showed major reduction when treated with all tested Ch-NPs concentrations, which followed a pattern based on concentration levels. The 1000 µL/L concentration of Ch-NPs completely stopped mycelial growth. The application of gamma irradiation (24 kGy) further enhanced the antifungal activity of Ch-NPs at each concentration level. The irradiated Ch-NPs at both 500 and 1000 µL/L achieved near-complete inhibition, outperforming their non-irradiated counterparts. The native chitosan treatments showed significantly reduced inhibitory effects at all tested concentrations, which demonstrated the enhanced bioactivity of nanoformulations, especially when combined with gamma irradiation, as shown in Fig. 7.

#### In vitro antagonistic activity of Trichoderma reesei and Bacillus subtilis against Fusarium oxysporum f. sp. sesami

The antagonistic potential of *Trichoderma reesei* and *Bacillus subtilis* against *F. oxysporum* f. sp. *sesami* was assessed on PDA medium. As illustrated in Table 1, both biocontrol agents significantly suppressed the pathogen’s mycelial growth compared to the untreated control. Among the two, *T. reesei* demonstrated the highest antifungal activity, with an inhibition percentage of 76.3%, whereas *B. subtilis* showed a moderate inhibition of 61.9%. These findings confirm the strong antagonistic capacity of *T. reesei* in suppressing FOS growth, likely due to its ability to produce hydrolytic enzymes and secondary metabolites.

**Table 1:** The percentage inhibition of mycelial growth of *F. oxysporum* f. sp. *sesami* caused by different biocontrol agents in Petri dish assays

Treatment	Inhibition (%) ± SE	Bioagents in petri dishes
*T. reesei*	76.3 ± 2.5	
*B. subtilis*	61.9 ± 2.1	
Control	0.0 ± 0.0	

The antagonistic activity of *Trichoderma reesei* and *Bacillus subtilis* against *Fusarium oxysporum* f. sp. *sesami* was evaluated on potato dextrose agar (PDA) medium under in vitro conditions. ± SE of three replicates.

#### Effect of different treatments on disease incidence (DI%) and disease severity (DS%) in infected plants

The effectiveness of biocontrol agents *Trichoderma reesei* and *Bacillus subtilis*, as well as the commercial fungicide Maxim-XL, was evaluated against Fusarium wilt caused by *Fusarium oxysporum* f. sp. *sesami* under greenhouse conditions. Results presented in Table 2 show that all treatments significantly reduced both disease incidence (DI%) and disease severity (DS%) compared to the infected control. *Trichoderma reesei* and Maxim-XL were equally effective in reducing disease incidence to 40%, while *B. subtilis* showed slightly less control (46.7%). Regarding disease severity, *T. reesei* provided the highest suppression (12%), followed by Maxim-XL (16%) and *B. subtilis* (20%). These reductions are statistically significant compared to the infected control (DI = 100%, DS = 82.7%) and fall within the calculated LSD values (DI: 9.7%, DS: 6.8%) (Table 2).

**Table 2:** Comparative efficacy of chitosan-based formulations and fungicide (Maxim) in managing Fusarium wilt of sesame

Treatment	Disease incidence % ± SE	Disease severity% ± SE
*T. reesei*	40 ± 3.5	12 ± 2.4
*B. subtilis*	46.7 ± 3.6	20 ± 2.5
Maxim-XL	40 ± 3.4	16 ± 2.3
Control infected	100 ± 0.0	82.7 ± 1.8
Control healthy	0.0 ± 0.0	0.0 ± 0.0
L.S.D value	9.7	6.8

± SE of three replicates. Statistical significance among treatments was determined by LSD test at *p* ≤ 0.05. Disease incidence and disease severity were assessed under controlled experimental conditions. *Trichoderma reesei* and *Bacillus subtilis* were applied as biological control agents. Maxim-XL was used as a chemical fungicide reference

#### Comparative efficacy of chitosan-based nanoparticles and chemical fungicide in reducing fusarium wilt incidence and severity in sesame

All tested treatments significantly reduced both disease incidence (DI%) and disease severity (DS%) compared to the infected control as shown in Table 3. Among the treatments, the fungicide Maxim showed the highest disease suppression (DI = 40.0%, DS = 12.0%), followed by gamma-irradiated chitosan nanoparticles (Ch-NPs) at 250 µL/L, which achieved a substantial reduction (DI = 46.7%, DS = 13.3%). Non-irradiated Ch-NPs (250–1000 µL/L) also demonstrated notable antifungal activity, though slightly less effective than their irradiated counterparts. Chitosan (non-nano) exhibited the weakest disease control, with DI% ranging from 60.0 to 73.3% and DS% between 28.0 and 37.3%. The infected control showed the highest disease levels (DI = 100%, DS = 82.7%), while the healthy control showed no disease symptoms. The control-infected plants exhibited minimal enzyme induction, while healthy plants maintained moderate baseline levels. Significant differences (*p* ≤ 0.05) among treatments were confirmed using LSD, indicating that both biocontrol agents and chemical treatment effectively enhanced host defense responses against Fusarium wilt.

**Table 3:** The comparative efficacy of different chitosan-based formulations and a chemical fungicide (Maxim) in managing Fusarium wilt in sesame plants

Treatment	Disease incidence (%) ± SE	Disease severity (%) ± SE	Relative efficacy description	Significance (LSD₀.₀₅)
Control healthy	0.0 ± 0.0	0.0 ± 0.0	No disease symptoms observed	—
Control infected	100.0 ± 0.0	82.7 ± 1.8	Highest disease incidence and severity	—
Maxim (fungicide)	40.0 ± 2.5	12.0 ± 1.9	Most effective treatment; strong disease suppression	Significant (*p* ≤ 0.05)
Ch-NPs 250 µL/L + 24 kGy	46.7 ± 2.7	13.3 ± 1.8	Highly effective; comparable to Maxim	Significant (*p* ≤ 0.05)
Ch-NPs 500 µL/L + 24 kGy	55.0 ± 2.8	25.0 ± 2.0	Moderate to strong antifungal activity	Significant (*p* ≤ 0.05)
Ch-NPs 1000 µL/L + 24 kGy	50.0 ± 2.6	23.0 ± 2.1	Effective but less than lower dose	Significant (*p* ≤ 0.05)
Ch-NPs 250 µL/L	66.0 ± 2.5	25.0 ± 2.2	Moderate effect; lower than irradiated form	Significant (*p* ≤ 0.05)
Ch-NPs 500 µL/L	45.0 ± 2.4	17.0 ± 1.9	Good suppression of disease symptoms	Significant (*p* ≤ 0.05)
Ch-NPs 1000 µL/L	45.0 ± 2.7	20.0 ± 2.0	Similar efficacy to 500 µL/L	Significant (*p* ≤ 0.05)
Chitosan 250 mg/L	73.3 ± 2.9	37.3 ± 2.3	Weak control; limited reduction in symptoms	Significant (*p* ≤ 0.05)
Chitosan 500 mg/L	68.0 ± 2.8	32.0 ± 2.1	Weak to moderate disease suppression	Significant (*p* ≤ 0.05)
Chitosan 1000 mg/L	60.0 ± 2.5	28.0 ± 2.0	Slight improvement over lower concentrations	Significant (*p* ≤ 0.05)

Disease incidence and disease severity were evaluated under controlled greenhouse conditions. Ch-NPs denote chitosan nanoparticles, and “24 kGy” indicates gamma irradiation at a dose of 24 kGy. Maxim was included as a chemical fungicide reference treatment. Values are expressed as mean ± standard error ± SE of three replicates. Statistical significance among treatments was determined by the LSD test at *p* ≤ 0.05

#### Induction of defense-selected enzyme activities in sesame plants treated with biocontrol agents and fungicide under Fusarium oxysporum f. sp. sesami infection

The study examined the effects of *Trichoderma reesei* and *Bacillus subtilis* and Maxim-XL treatments on the activities of peroxidase (PO) and polyphenol oxidase (PPO) and chitinase and phenylalanine ammonia-lyase (PAL) in sesame plants under greenhouse conditions. The plants that received Maxim-XL treatment produced the most enzymatic activity levels, which included chitinase at 68.5 U/g FW and increased PPO and PO levels. The plants that received T. reesei treatment exhibited elevated levels of PO and PPO and chitinase activities when compared to the infected control group.

#### Induction of defense-related enzymes in sesame plants treated with chitosan-based formulations and fungicide under Fusarium oxysporum infection

**Fig. 8:**
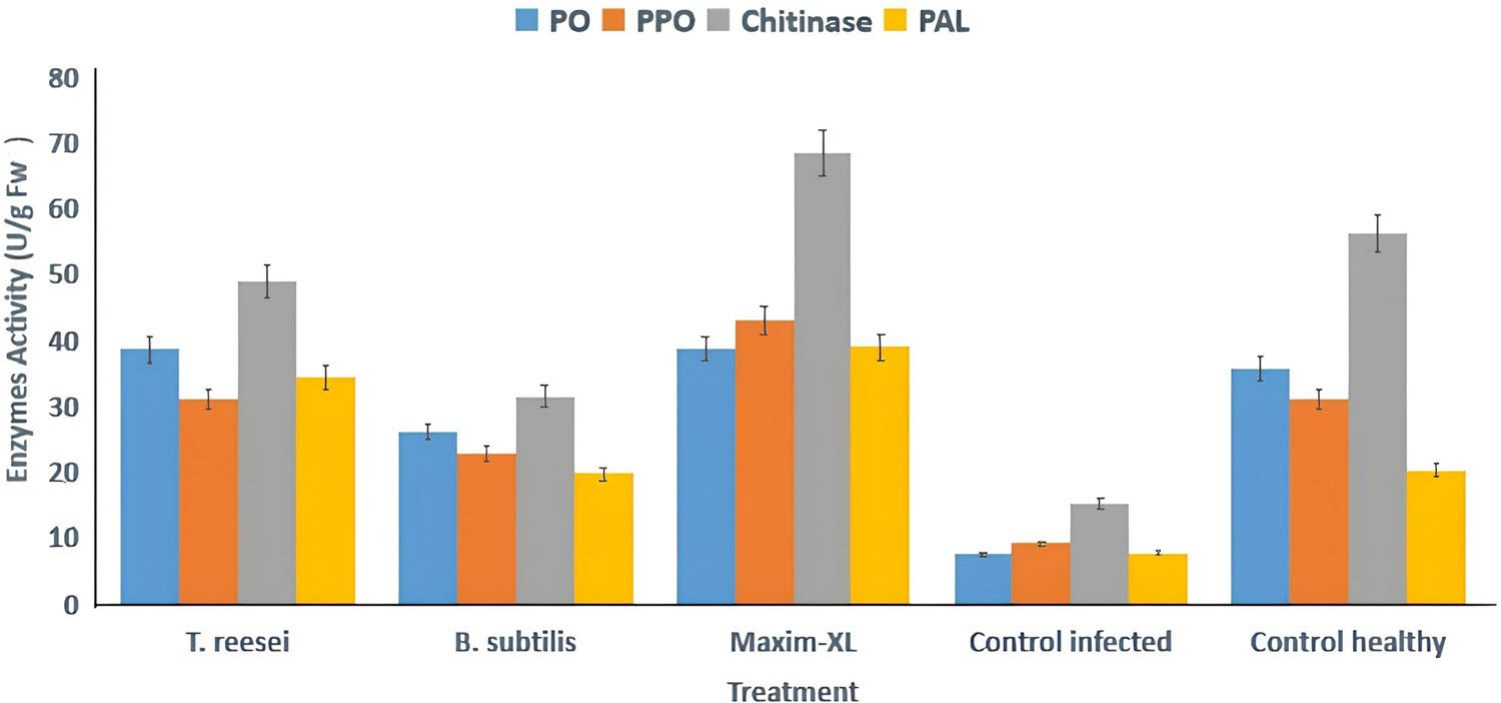
Biocontrol agents and fungicide on defense-related enzyme activities in sesame plants infected with *Fusarium oxysporum* f. sp*. sesami.* Activities of peroxidase (PO), polyphenol oxidase (PPO), chitinase, and phenylalanine ammonia-lyase (PAL) were measured in leaves of sesame plants treated with *Trichoderma reesei*, *Bacillus subtilis*, or the fungicide Maxim-XL, compared with infected and healthy controls. Data represent mean enzyme activity (U/g FW) ± standard error (SE) of three independent replicates. Significant differences among treatments were determined using LSD at *p* ≤ 0.05

The effects of native chitosan (250–1000 mg/L), chitosan nanoparticles (ChNPs, 250–1000 µL/L), and gamma-irradiated ChNPs (24 kGy) were compared to Maxim and controls on the enzymatic activity of peroxidase (PO), polyphenol oxidase (PPO), chitinase, and phenylalanine ammonia-lyase (PAL) in sesame plants. Maxim and irradiated ChNPs at 250 µL/L showed the highest induction of all enzymes, particularly chitinase and PAL. Control infected plants exhibited minimal enzyme activity, while healthy plants maintained moderate baseline levels. The elevation in enzyme activity indicates the elicitation of plant defense responses, supporting the potential of nano-enabled chitosan treatments as eco-friendly inducers of systemic resistance (Fig. 8).

**Fig. 9:**
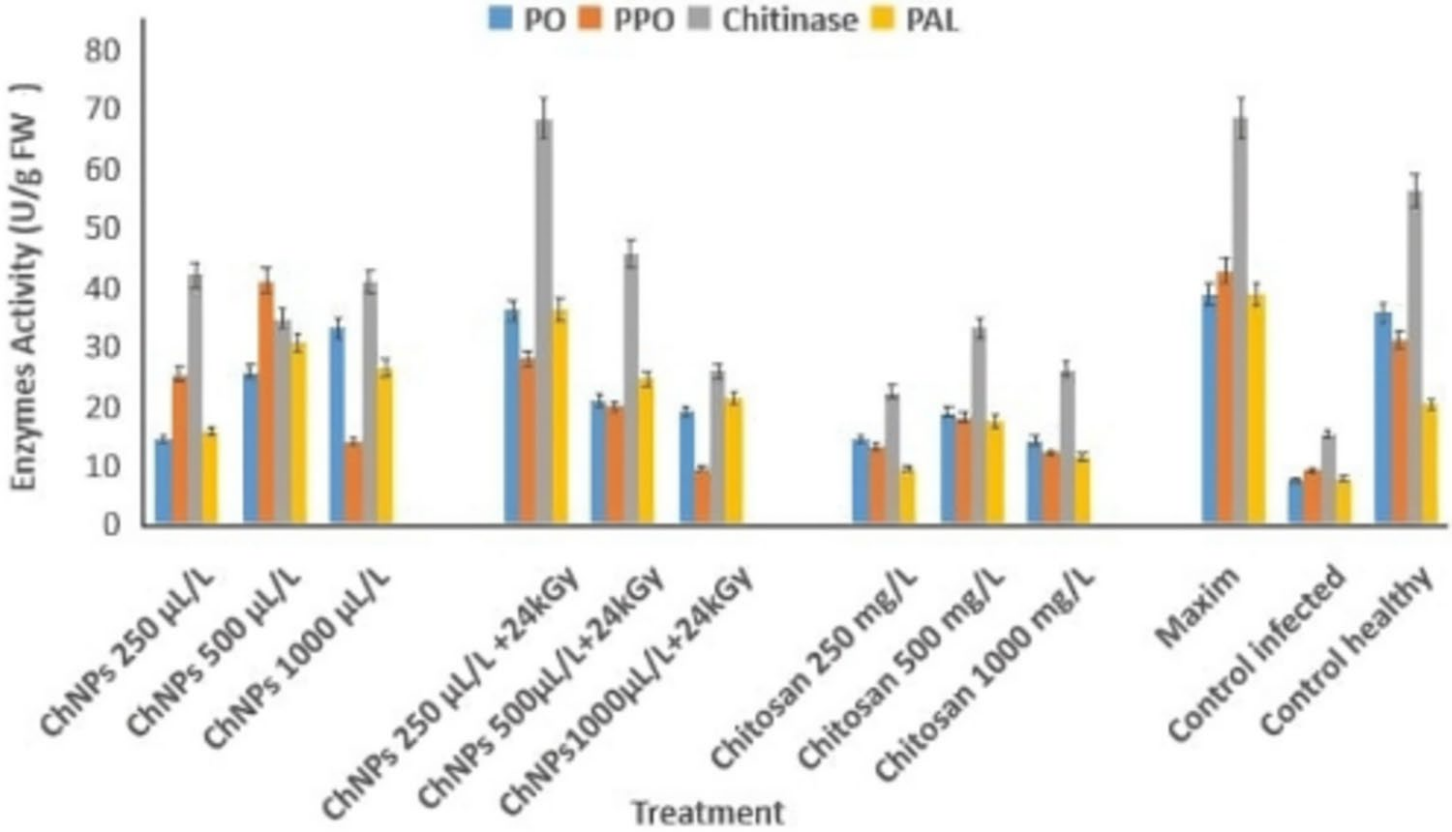
Effect of chitosan-based treatments and fungicide on defense-related enzyme activities in sesame plants. The figure illustrates the activities of four defense enzymes—peroxidase (PO), polyphenol oxidase (PPO), chitinase, and phenylalanine ammonia-lyase (PAL)—in sesame plants treated with different concentrations of chitosan nanoparticles (ChNPs), gamma-irradiated ChNPs, pure chitosan, and the chemical fungicide Maxim, compared with infected and healthy controls. Each bar represents the mean of three biological replicates ± standard error. Enhanced enzyme activities, particularly in treatments involving irradiated ChNPs and Maxim, indicate the activation of plant defense mechanisms against *Fusarium oxysporum* f. sp. *sesami*. Data represent mean enzyme activity (U/g FW) ± standard error (SE) of three independent replicates. Significant differences among treatments were determined using LSD at *p* ≤ 0.05

Chitosan-based treatment alongside fungicide showed significant enhancement of defense enzymes and activities in sesame plants that suffered from *Fusarium oxysporum* f. sp. *sesami* infection. Peroxidase (PO), polyphenol oxidase (PPO), and chitinase, along with phenylalanine ammonia-lyase (PAL) defense enzymes, were measured in fresh leaf tissues at (U/g FW) concentrations after applying chitosan nanoparticles (ChNPs) at 250, 500, and 1000 µL/L and irradiated ChNPs at 24 kGy and bulk chitosan at 250, 500, and 1000 mg/L and Maxim fungicide to healthy and infected plants. The defense enzyme production peaked in irradiated ChNPs at 250 µL/L + 24 kGy treatment, followed by an enhanced plant defense response from fungicide application against Fusarium wilt based on Fig. 9.

The results show clear and statistically meaningful differences among the applied treatments. All treatments produced substantial decreases in disease occurrence and intensity when compared to the infected control group, according to LSD values of 21.7% and 6.4%. The results showed Maxim-XL and Ch-NPs 250 µL L⁻^1^ + 24 kGy achieved the best disease control because their percentages fell under the LSD threshold. The treatments produced better plant growth and higher seed production which indicates their impact went beyond disease control to enhance total plant performance. The infected control showed poor results across every parameter when compared to the moderate improvements produced by chitosan 1000 mg L⁻^1^ and *B. subtilis*. The statistical analysis showed that nano-enabled and biological treatments functioned as effective alternatives to synthetic fungicides because multiple treatments delivered results that either matched or exceeded those of Maxim-XL (Table 4).

**Table 4:** Impact of selected treatments on disease parameters and growth of sesame plants infected with *Fusarium oxysporum* f. sp. *sesami* under field conditions

Treatment	Disease incidence%	Disease severity%	Plant height (cm)	Number of capsules per plant	Seed yield per plant (g)
Ch-NPs 250 µL/L + 24 KG	46.7	16.0	265.0	293.3	39.2
Ch-NPs 500 µL/L	53.3	18.7	255.0	260.0	36.0
Chitosan 1000 mg/L	66.7	29.3	227.3	186.0	31.3
*T. reesei*	46.7	17.3	265.0	235.7	38.3
*B. subtilis*	60.0	22.7	205.3	224.7	35.0
Maxim-XL	46.7	13.3	271.0	262.7	38.7
Control infected	100.0	81.3	165.0	61.7	20.0
L.S.D at 5%	21.7	6.4	10.2	56.6	5.0

Nano-chitosan formulations, biological agents, and chemical fungicide on disease incidence, disease severity, and yield-related traits of sesame plants infected with Fusarium spp. Values represent the mean of replicated treatments. Differences among treatments were evaluated using LSD at the 5% probability level

## Discussion

Sesame wilt caused by *Fusarium oxysporum* f. sp. *sesami* is among the most serious diseases, with potential for large yield losses in oilseed production systems (Farhaoui et al. 2025). This study provides compelling evidence that gamma-irradiated chitosan nanoparticles (Ch-NPs) can effectively suppress *Fusarium oxysporum* f. sp. *sesami*, the causative agent of sesame wilt, through a dual mode of action that involves both direct antifungal effects and induction of host defenses when applied alone or in combination with *Trichoderma reesei*. The findings imply that while biological control agents, especially *T. reesei*, further increase disease inhibition and promote plant growth in greenhouse and field settings, nanosizing and gamma irradiation boost the antifungal activity of chitosan. Chitosan’s antifungal activity is explained by its capacity to damage fungal cell walls and membranes, causing structural and morphological changes as well as molecular disarray in fungal cells (Dhlamini et al. 2024; López-Moya et al. 2019).

In field trials, the combined application of gamma-irradiated Ch-NPs (24 kGy, 250 µL L⁻^1^) and *T. reesei* achieved comparable disease control to the chemical fungicide Maxim-XL, while simultaneously increasing plant height, capsule number, and seed yield, reflecting both pathogen suppression and enhanced plant vigor.

The application of gamma irradiation effects on Ch-NPs. 89.08–113.63 nm of particle size before irradiation reduced to 48.11–56.22 nm of particle size after irradiation. Thus, improved dispersion and reactivity on the surface and solubility resulted in stronger electrostatics with the negative fungal cell wall and resulted in disruption of the membrane and leakage of the cytoplasm of the cell (Muley et al. 2019; Yan et al. 2021; Akdaşçi et al. 2025).

As the molecular structure and biological activity were more mobile during the fungal activity, depolymerization partially resulted in improved antimicrobial activity. Out of 1000 µL L⁻^1^ of irradiated Ch-NPs, there was a zero suppression of *F. oxysporum* growth, which depicts the synergy of irradiation and nanoscale action. Ironically, the polysaccharide chitosan assists in binding the important metal and biological substances, fungal metabolism, and better absorption into plant and fungal tissue biofilms, which amplifies the antimicrobial activity (Youssef et al. 2019; Saharan et al. 2013; Solis Vizcaino et al. 2024).

Along with this nano-enabled protection, biological control agents take effect. Among the tested strains, based solely on in vitro antagonism, *T. reesei* overcame *Bacillus subtilis* by suppressing 76% of the fungus in vitro. This also manifested in significant suppression of the diseases in terms of incidence and severity in vivo. This increased activity was probably associated with Johann’s findings about the secretion of hydrolytic enzymes, chitinases, β−1,3-glucanases, and proteases involved in the degradation of pathogens’ cell walls and further secondary metabolites’ detoxification, antifungal, and plant growth promotion (Damodaran et al. 2020; Manzar et al. 2022; Yao et al. 2023; Wonglom et al. 2024).

The activities of defense enzymes, such as peroxidase (PO), polyphenol oxidase (PPO), chitinase, and phenylalanine ammonia-lyase (PAL), were greatly increased during the defense response of the sesame plants, thereby exhibiting systemic resistance. These enzymes also strengthen the cell walls, provide enhanced detoxification of the harmful pathogen metabolites, and improve the cell’s antimicrobial phenolic response (Li and Zhu 2013; Naguib et al. 2021).

The increase in PAL and chitinase activity positively correlates to the increase in lignin production and the breakdown of the fungal cell wall due to the strong engagement of the structural and biochemical defenses. Hence, the mixture of gamma-irradiated Ch-NPs and *T. reesei* presents not only two-pronged disease containment, by inhibiting the pathogen directly and inducing immune sensitization in the host, but also serves as a sustainable and thoroughly defendable substitute for the conventional fungicides.

## Conclusion

The present study provides clear evidence that gamma-irradiated chitosan nanoparticles offer a promising and practical path toward more sustainable management of sesame wilt. By subjecting the nanoparticles to gamma irradiation, their structural and functional properties were noticeably refined, resulting in a formulation with stronger antifungal capacity and a more consistent ability to activate host defense mechanisms. These improvements were reflected across all levels of evaluation: the pathogen was nearly fully suppressed in vitro, and both greenhouse and field experiments showed disease reductions comparable to those achieved with the commercial fungicide Maxim-XL. Gamma-irradiated chitosan nanoparticles, alone or synergistically with *Trichoderma reesei*, effectively suppressed sesame wilt while enhancing plant growth, capsule development, and seed yield. The novelty of this research is that the use of gamma irradiation enhances the bioproperties of chitosan nanoparticles which, working together with the fungus *Trichoderma reesei*, provide an enhanced plant defense response through increased levels of certain important plant defense enzymes (PO, PPO, and Chitinase, and PAL) that directly suppress plant pathogen development, therefore inducing greater systemic immunity for increased durability against future attack. Consequently, the method is environmentally responsible and based on scientifically valid and sustainable methods rather than conventional chemical fungicidal products.

## Data Availability

All data supporting the findings of this study are available upon request.
